# Supplementary material for: SOD3 improves the tumor response to chemotherapy by stabilizing endothelial HIF-2α
Source: Nat Commun. 2018 Feb 8;9:575. doi: 10.1038/s41467-018-03079-1 (PMC5805714; doi:10.1038/s41467-018-03079-1)
Supplement: Supplementary file 1 — Supplementary Information [file 41467_2018_3079_MOESM1_ESM.pdf]

Supplementary Information

SOD3 improves the tumor response to chemotherapy by stabilizing endothelial HIF-2α

Mira et al.

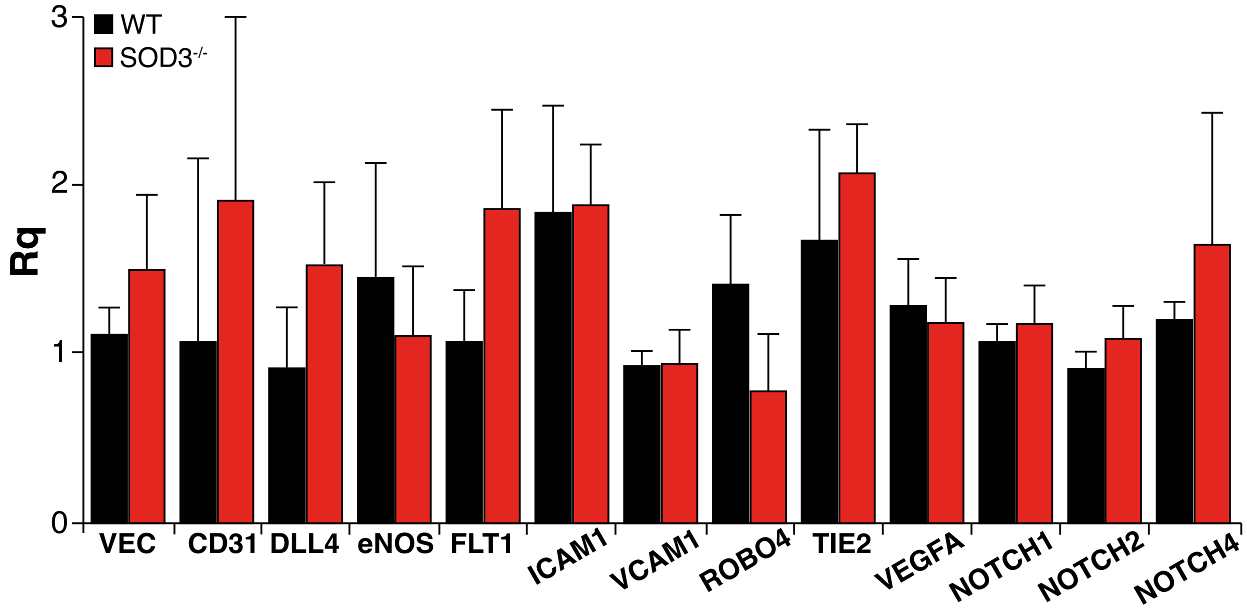

Supplementary Figure 1. Analysis of the vasculature of WT and SOD3$^{-/-}$ mice in physiological conditions. mRNA levels of indicated EC markers, quantified by RT-qPCR in primary EC purified from lungs of WT and SOD3$^{-/-}$ mice. Data shown as mean ± SEM of triplicates from three independent experiments. None of the markers analyzed showed statistical differences among the genotypes (two-tailed Student's *t*-test).

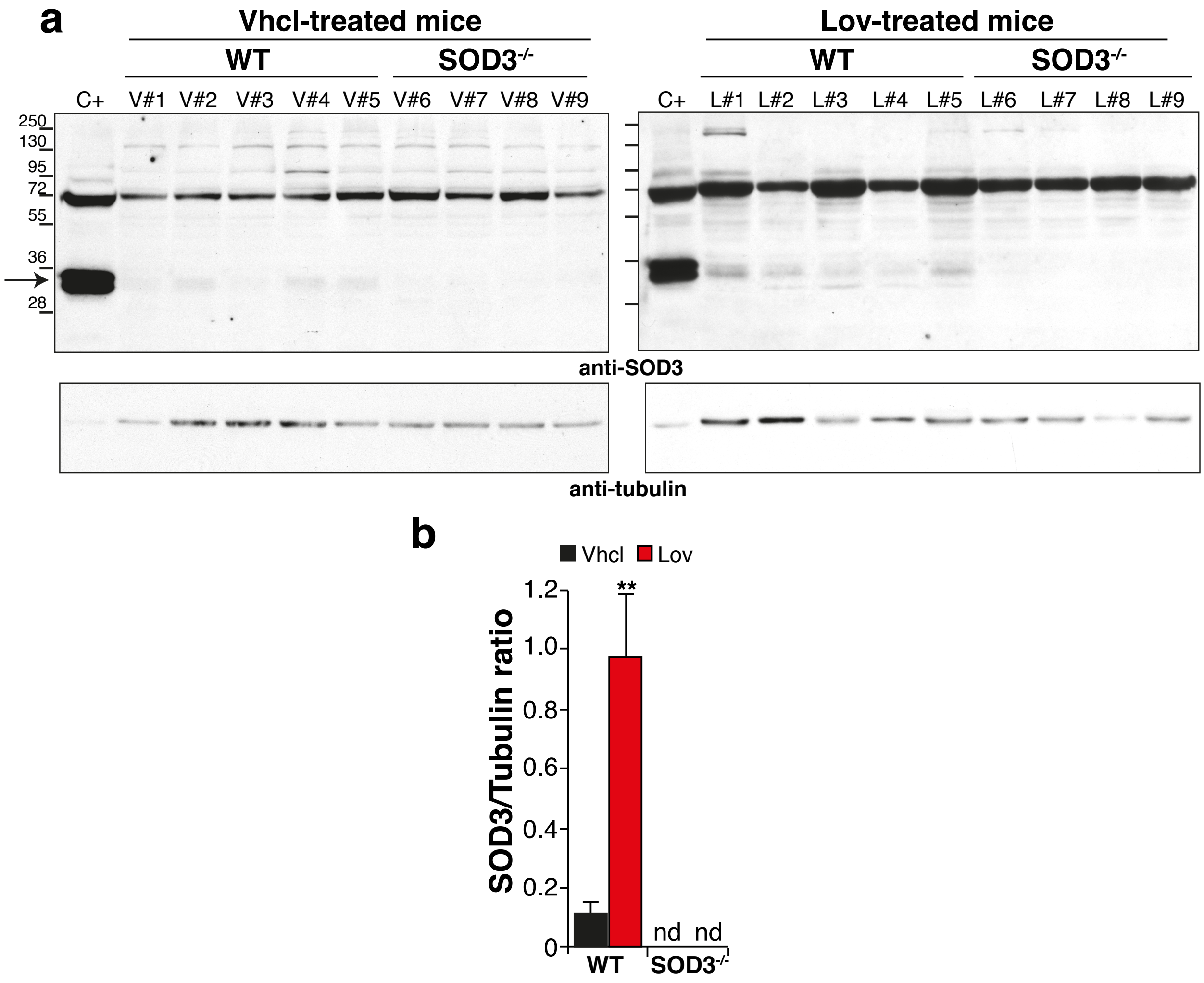

Supplementary Figure 2. Lovastatin treatment induces SOD3 upregulation in LLC tumors. (**a**) Immunoblot analysis of extracts of representative LLC tumors grown in Vhcl- or Lov-treated WT or $SOD3^{-/-}$ mice, using anti-SOD3 (top) or anti-tubulin antibodies (bottom; loading control). C+ is a lung tissue extract used as positive control for SOD3. (**b**) The intensity of SOD3- and tubulin-specific bands was measured by densitometry, and the ratio calculated for individual tumor samples. The graph shows mean ± SEM of the SOD3/tubulin ratio in Vhcl- and Lov-treated mice (n = 10/group); nd, not detected. ** $p < 0.01$, two-tailed Student's *t*-test.

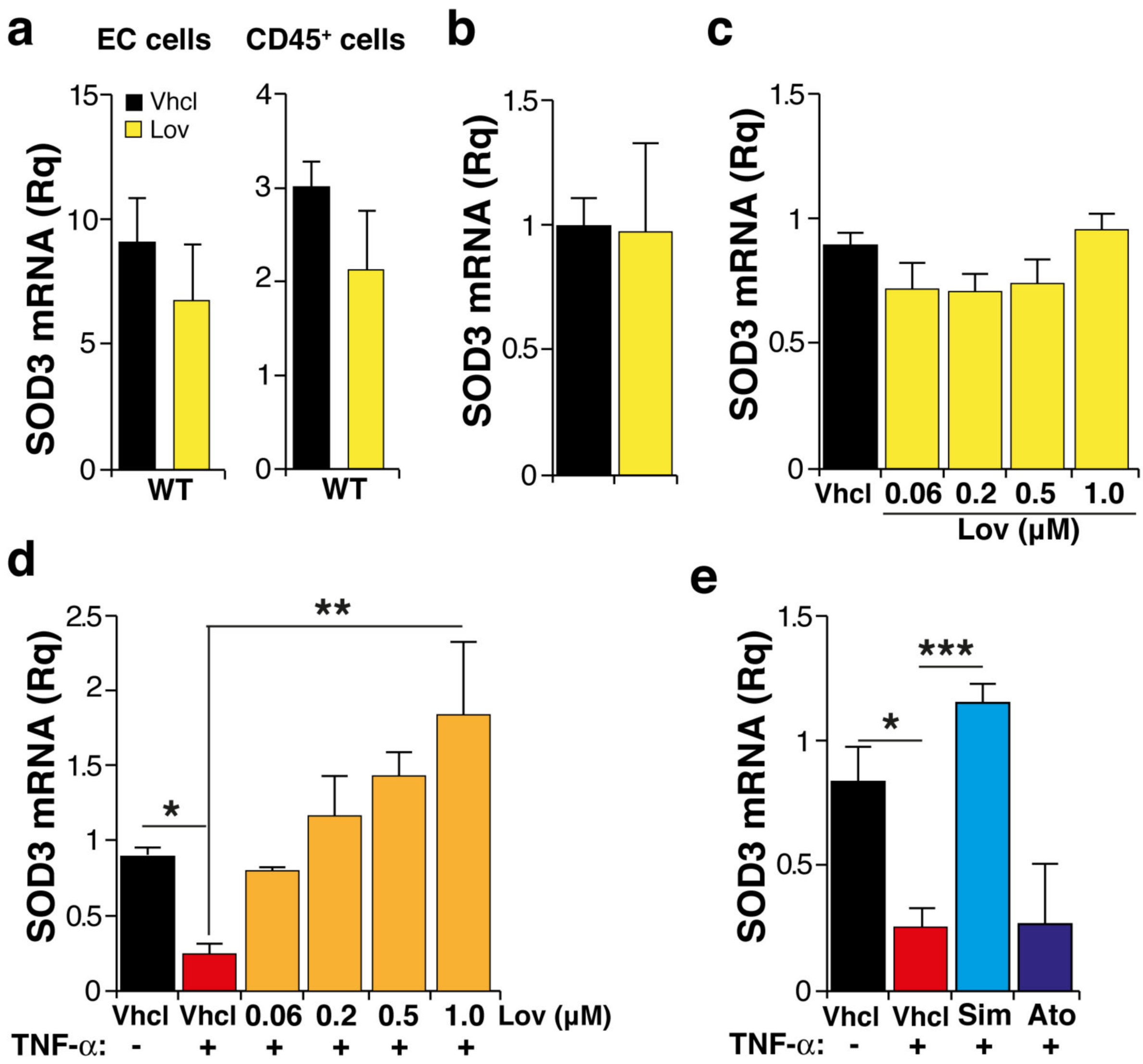

Supplementary Figure 3. Lov induces SOD3 through an indirect mechanism. (**a**) Relative SOD3 mRNA levels in endothelial cells isolated from lung and in leukocytes isolated from spleen of tumor-free mice treated with Vhcl or Lov as indicated. Data are mean ± SEM of triplicates (n = 4). Values were not statistically different; two-tailed Student's *t*-test. (**b**) Effect of Lov (1 μM) on SOD3 mRNA levels in 1G11 cells. Differences were not significant; two-tailed Student's *t*-test. (**c**) Effect of different Lov doses on SOD3 mRNA levels in 3T3 cells. Values were not statistically different; one-way ANOVA with Dunnet's post-hoc test, using Vhcl as reference. (**d**) 3T3 cells were incubated for 48 h with TNF-α and the indicated doses of Lov or the corresponding Vhcl (see Methods); SOD3 mRNA levels were determined by RT-qPCR. (**e**) 3T3 cells were incubated (48 h) with TNF-α and either simvastatin (Sim) or atorvastatin (Ato), or their corresponding Vhcl. For *b-e*, data are mean SEM of triplicates (*n* = 3). * $p < 0.05$, ** $p < 0.01$, one-way ANOVA with Tukey's post-hoc test.

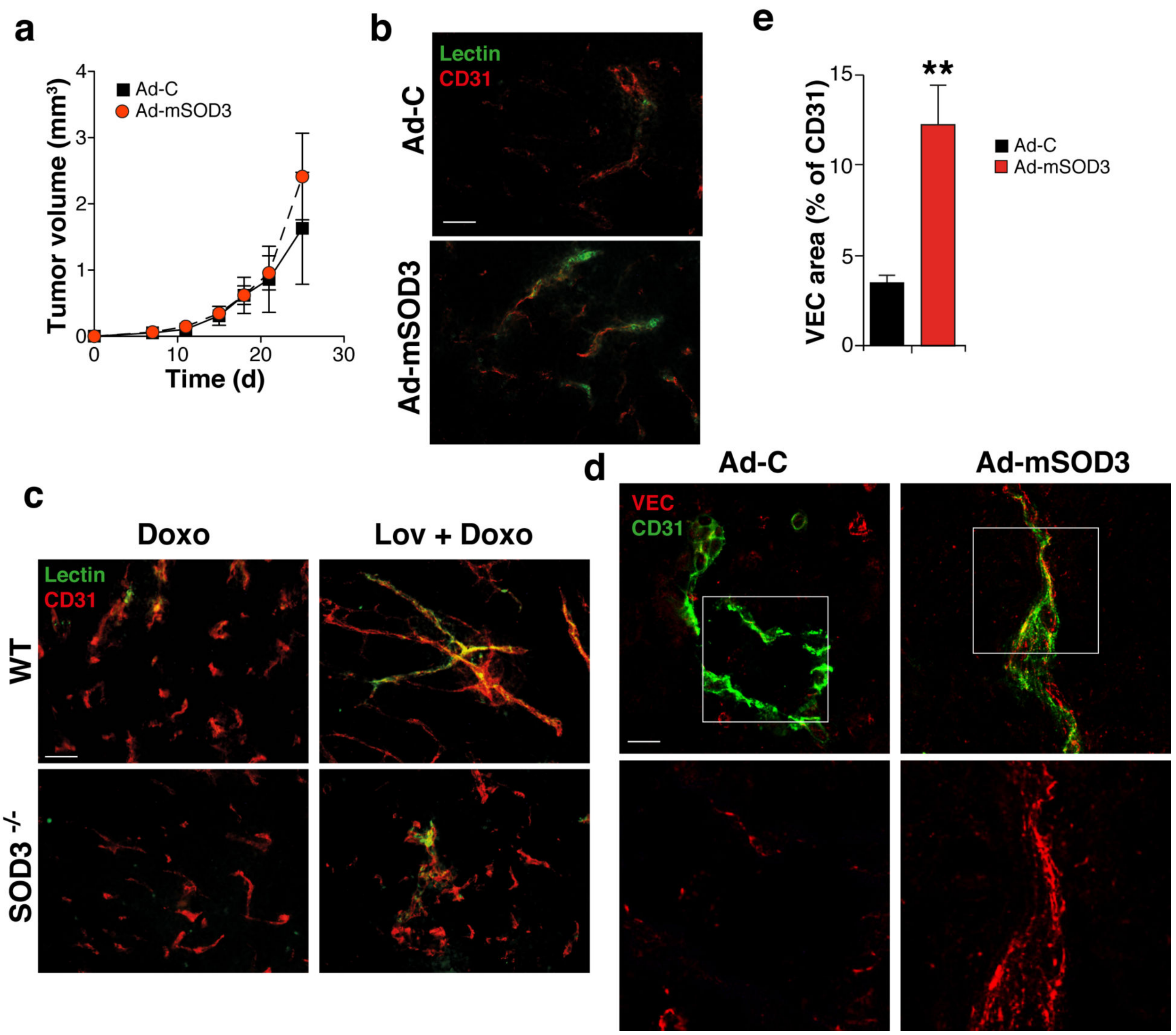

Supplementary Figure 4. Characteristics of tumor blood vessels after SOD3 upregulation. (**a**) Growth kinetics of Ad-C- or Ad-mSOD3-injected LLC tumors as in Fig. 1c, but without Doxo treatment. Values were not statistically different; two-tailed Student's *t*-test. (**b**) Representative immunofluorescence images of FITC-lectin-perfused (green) and CD31-stained (red) vessels of Ad-C- and Ad-mSOD3-treated tumors. (**c**) Representative immunofluorescence images of FITC-lectin-perfused (green) and CD31-stained (red) vessels of Vhcl+Doxo- or Lov+Doxo-treated tumors grown in WT and SOD3-/- mice. (**d**) Representative immunofluorescence images from Ad-C- and Ad-mSOD3-treated tumors stained for CD31 (green) and VEC (red). Top panels, merged images; bottom, VEC staining in a magnified area. (**e**) Quantification of images as in d. Data shown as mean ± SEM (n = 20 images from ≥5 mice/group); ** p <0.01 two-tailed Student's *t*-test. Bar, 50 μm.

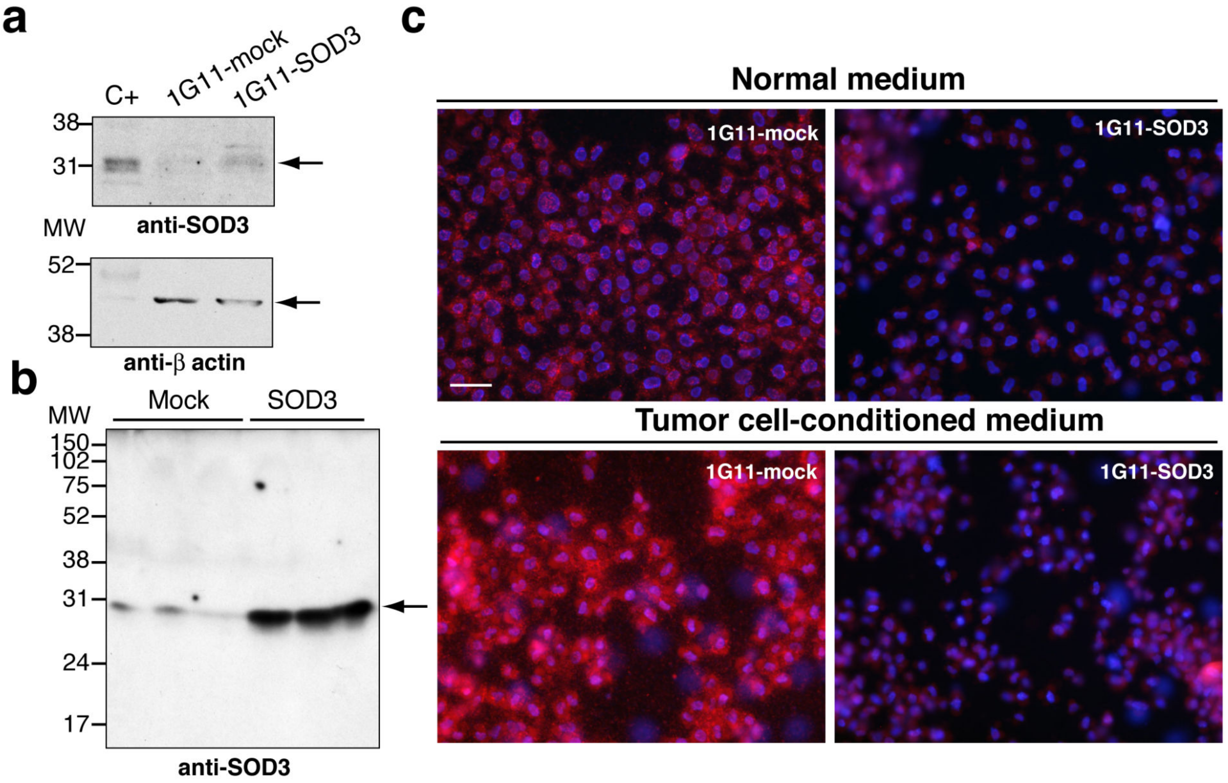

Supplementary Figure 5. Characterization of 1G11-SOD3 cells. (**a**) Lysates prepared from 1G11-mock and 1G11-SOD3 cells were resolved by SDS-PAGE, transferred and immunoblotted sequentially with anti-SOD3 and -β-actin (loading control) antibodies. C+, kidney extract used as SOD3 reference. (**b**) Immunoblot of conditioned medium from cells as above, using anti-SOD3 antibodies. (**c**) 1G11-mock and SOD3 cells in exponential growth were cultured in normal medium (top) or exposed to conditioned medium from a tumor cell line (2 h; bottom). Cultures were stained with the cell-permeable superoxide probe dihydroethidium, which becomes red after oxidation; nuclei were DAPI-counterstained (blue). Bar, 20 μm

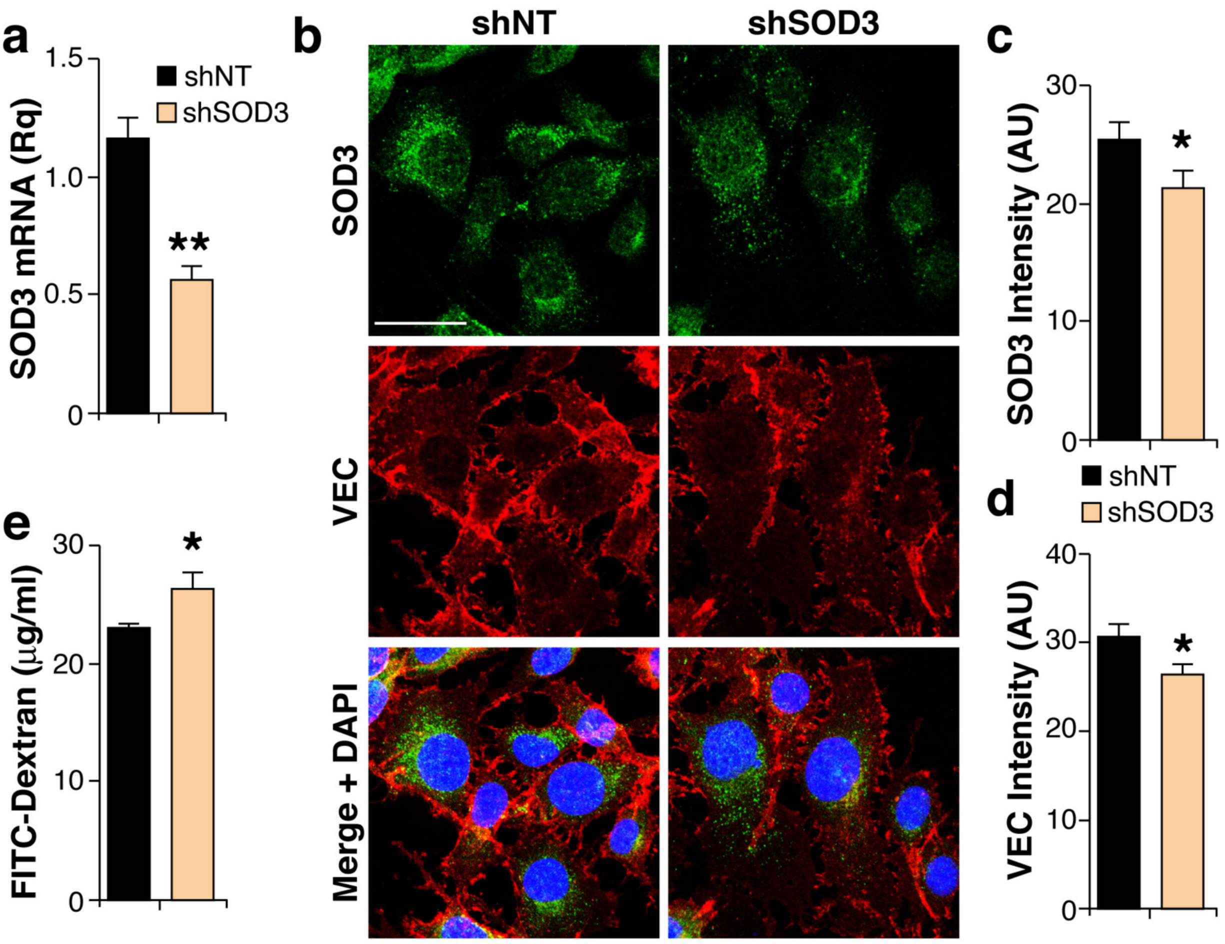

Supplementary Figure 6. SOD3 silencing decreases VEC expression and increases 1G11 cell permeability. (**a**) Relative SOD3 mRNA levels in 1G11 expressing control (shNT) and SOD3-specific shRNA. Data shown as mean ± SEM of triplicates (n = 3). (**b**) Representative images of shNT- and shSOD3-transduced 1G11 cells stained for SOD3 (green) and VEC (red). The merge of both chanels with DAPI counterstaining of nuclei (blue) is shown at bottom. (**c, d**) Quantification of the mean fluorescence intensity of SOD3 (*c*) and VEC (*d*) in cells from images as in *b*. Data shown as mean ± SEM (*n* = 43 and 49, for shNT and shSOD3-transduced cells). (**e**) FITC-dextran (40 kDa) permeability of 1G11-shNT and -shSOD3 monolayers. Data are mean ± SEM of triplicates (*n* = 3). $^*p < 0.05$, $^{**}p < 0.01$, two-tailed Student's *t*-test. Bar, 20 μm.

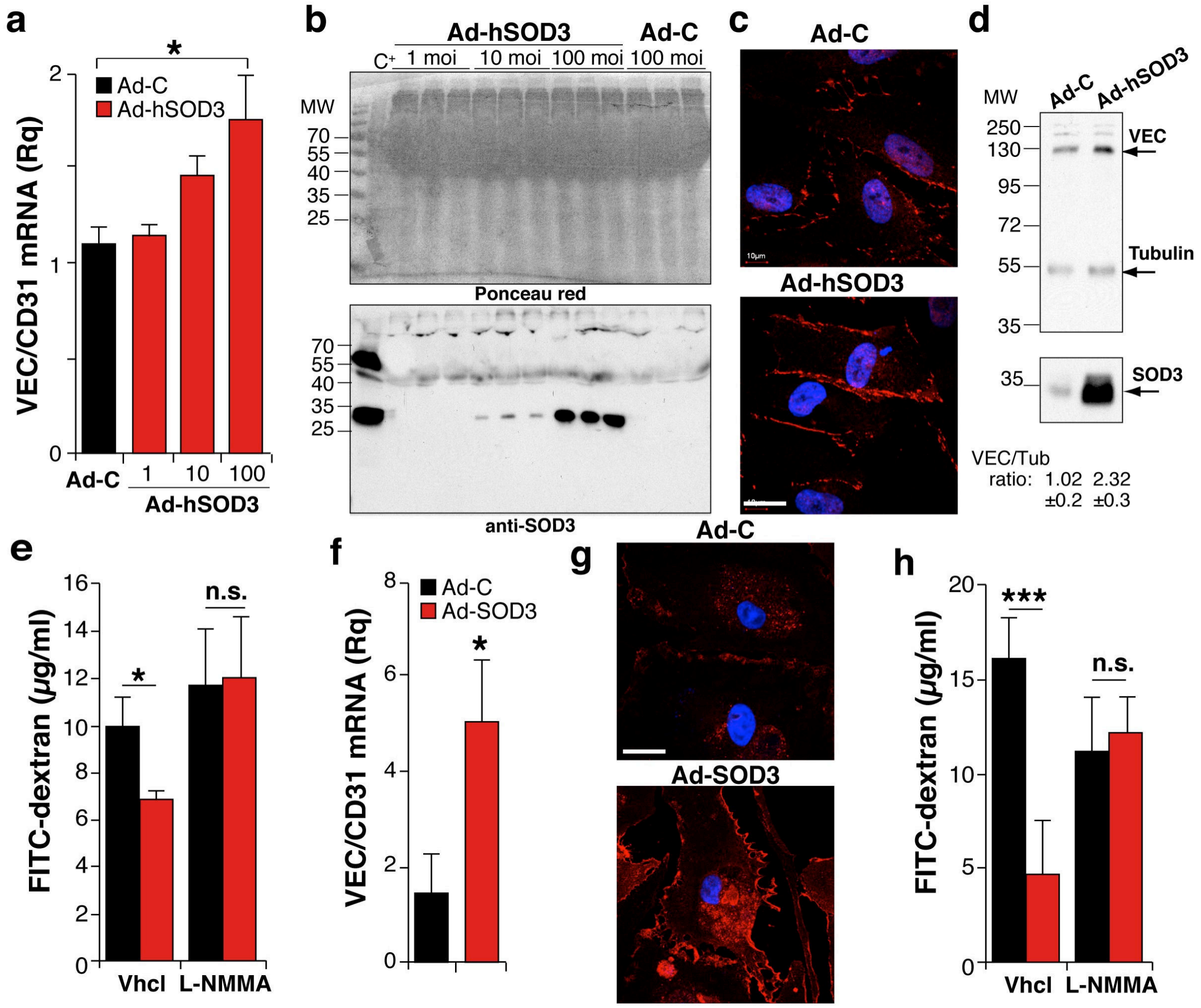

Supplementary Figure 7. SOD3 induces VEC transcription and reduces permeability in human and bovine EC. (**a**) VEC mRNA levels quantified by RT-qPCR in HDMEC cells infected with adenovirus encoding hSOD3 (1, 10 or 100 m.o.i.) or β-galactosidase (Ad-C, 100 m.o.i.). Data shown as mean ± SEM of triplicates (*n* =3). * p <0.05, ANOVA with Dunnett's post-hoc test, using Ad-C as reference. (**b**) Conditioned medium from the cells in A. Top, Ponceau red staining; bottom, immunoblot of the same filter with an anti-SOD3 antibody. C+, lung extract used as reference for SOD3 (*n* = 3). (**c**) Conditioned medium from the cells in *a*. Top, Ponceau red staining; bottom, immunoblot of the same filter with an anti-SOD3 antibody. C+, lung extract used as reference for SOD3 (*n* = 3). (**c**) Immunofluorescence of Ad-C- or Ad-hSOD3-transduced HDMEC cells (both 100 m.o.i.) stained with anti-VEC antibody; nuclei are DAPI-counterstained (blue). Representative cells of ≥250 analyzed (*n* = 3). (**d**) Immunoblot of lysates from Ad-C- and Ad-SOD3-infected HDMEC cells, using anti-VEC, -tubulin (loading control) and -hSOD3 antibodies (*n* = 3). The VEC/tubulin ratio determined by densitometry is indicated at bottom (mean ± SEM). (**e**) FITC-dextran (40 kDa) permeability of Ad-C- and Ad-hSOD3-infected HDMEC monolayers, treated with Vhcl or the pan-NOS inhibitor L-NMMA (*n* = 3). (**f**) VEC mRNA levels in Ad-SOD3- or Ad-C-infected BAEC (both 100 m.o.i.). Data shown as mean ± SEM (*n* = 3). (**g**) Immunofluorescence of BAEC transduced with Ad-C or Ad-hSOD3, and stained with anti-VEC antibody; nuclei are DAPI-counterstained (blue). n ≥ 250 cells. (**h**) Dextran permeability of Ad-C- and Ad-hSOD3-infected BAEC monolayers, treated with Vhcl or L-NMMA (*n* = 3). For *e*, *f*, *h*, * p <0.05, *** p <0.001, two-tailed Student's t-test; n.s., not significant. Bar, 20 μm.

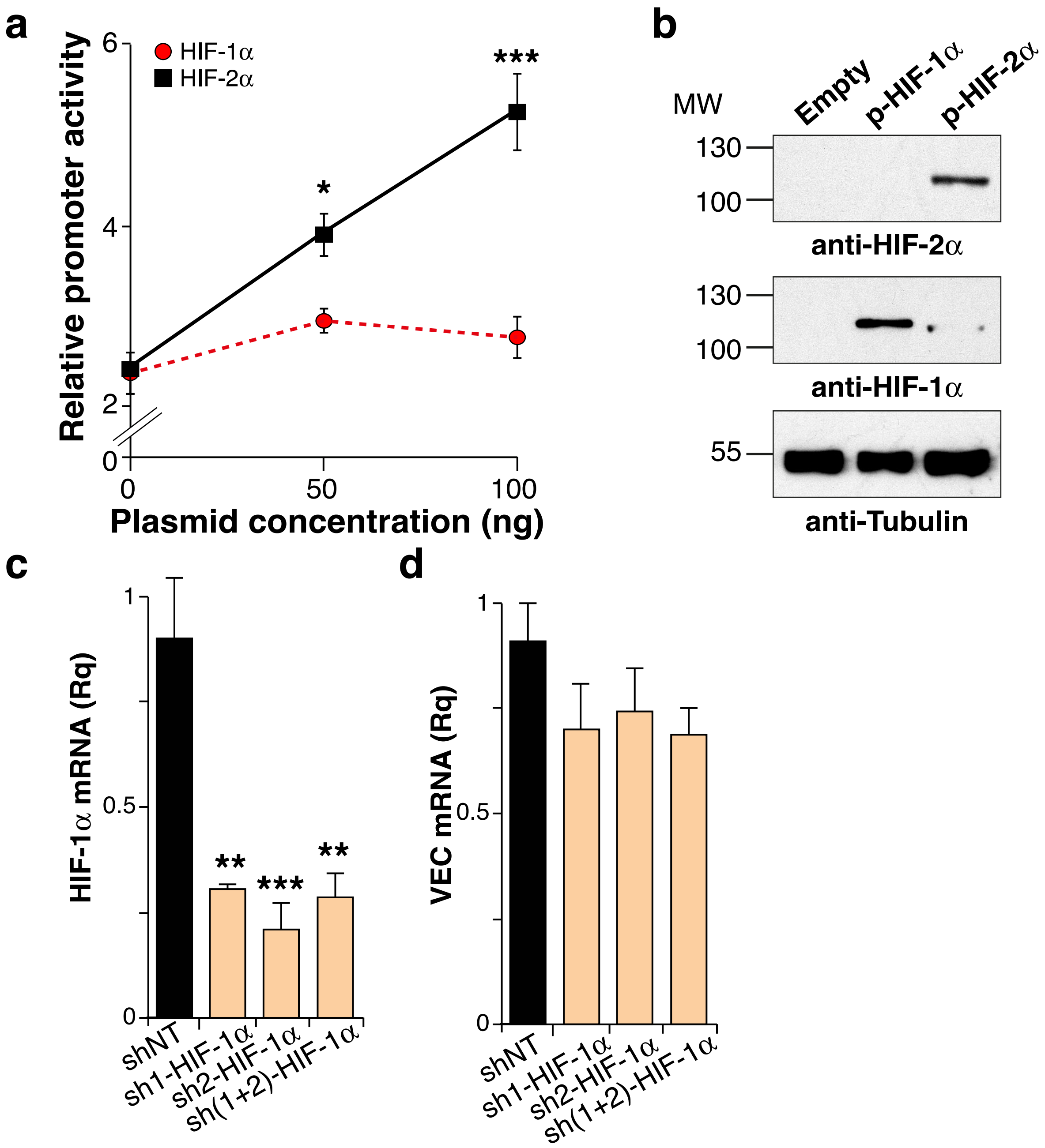

Supplementary Figure 8. HIF-2α, but not HIF-1α, induces VEC transcription. (**a**) 3T3 cells were transfected with an empty vector or various doses of a plasmid encoding murine HIF-1α or HIF-2α, as well as the VEC promoter-Luc and pSV40-renilla reporter plasmids. Relative VEC promoter activity was calculated as indicated (Methods). Data shown as mean ± SEM of triplicates from two independent experiments. * $p < 0.05$; *** $p < 0.001$, one-way ANOVA with Dunnett's post-hoc test, using empty vector-transfected cells as reference. (**b**) Immunoblot analysis of extracts of from the cells as in a, using anti-HIF-2α, anti-HIF-1α, or anti-tubulin antibodies (bottom; loading control). (**c, d**) HIF-1α silencing does not affect VEC levels in 1G11 cells. 1G11 cells were transduced with retroviruses encoding control (shNT), two distinct short hairpin RNA targeting HIF-1α, or a mixture of the two sh HIF-1α RNA. The mRNA level for HIF-1α (*c*) and VEC (*d*) was determined in puromycin-selected cells, using that of shNT-transduced cells as reference. ** $p < 0.01$; *** $p < 0.001$, one-way ANOVA with Dunnett's post-hoc test. Similar statistical analyses of VEC mRNA levels showed no significant differences (shNT vs sh1-HIF-1α, $p = 0.17$; shNT vs sh2-HIF-1α, $p = 0.27$; shNT vs sh1+2-HIF-1α, $p = 0.14$).

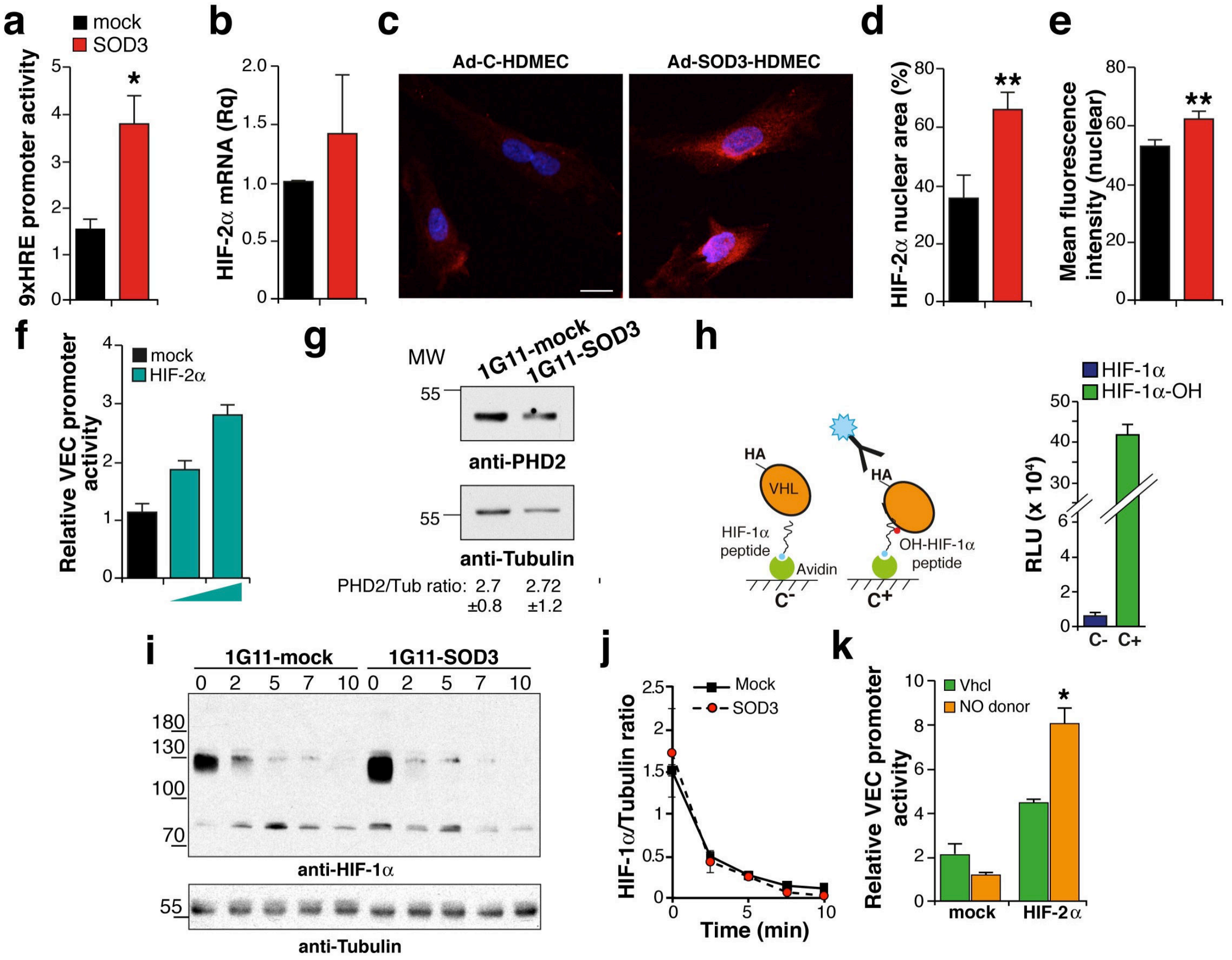

Supplementary Figure 9. SOD3 and NO regulate HIF-2α stability and transcriptional activity. (**a**) Relative activity of a 9xHRE synthetic promoter in mock- or SOD3-transfected 1G11 cells (*n* = 3). (**b**) Basal HIF-2α mRNA levels in mock- or SOD3-transfected 1G11 cells (*n* = 4). (**c**) Representative images of HIF-2α staining in Ad-C- and Ad-SOD3-infected HDMEC cells (n ≥250 cells/condition). (**d**, **e**) Percentage of nuclear area and mean fluorescence intensity in images as in *c*. (**f**) Relative VEC promoter activity in 3T3 cells transfected with increasing quantities of HIF-2α plasmid (green bars). An empty plasmid was used as control (black bar; *n* = 3). (**g**) Immunoblot analysis of lysates from 1G11-mock and -SOD3 cells with anti-PHD2 and -tubulin (loading control) antibodies; the ratio (mean ± SEM) is indicated at bottom (*n* = 3). (**h**) Diagram of the ELISA designed to analyze specificity of recombinant-produced, HA-tagged VHL protein (left). The graph shows the result of a representative ELISA (right). HA-VHL binding to the hydroxylated HIF-1α peptide is >40-fold higher than the non-hydroxylated peptide. RLU, relative luciferase units. (**i**) Representative immunoblot of HIF-1α levels after re-oxygenation of cycloheximide-treated 1G11-mock and -SOD3 cells cultured in hypoxia; tubulin was used as loading control. (**j**) HIF-1α/tubulin ratio obtained by densitometric quantification of immunoblots as in *i* (*n* = 3). No significant differences were observed between cell lines; two-way ANOVA with Bonferroni post-hoc correction. (**k**) 3T3 cells were cotransfected with HIF-2α (or empty vector as control) and the VEC promoter, and incubated with DETA-NONOate (NO donor) or Vhcl; the relative luciferase activity driven by the VEC promoter was then measured (*n* = 5). (*a*, *b*, *d*, *e-h*, *j*, *k*) Data shown as mean ± SEM of triplicates; * p <0.05, ** p < 0.01, two-tailed Student's t-test. Bar, 20 μm.

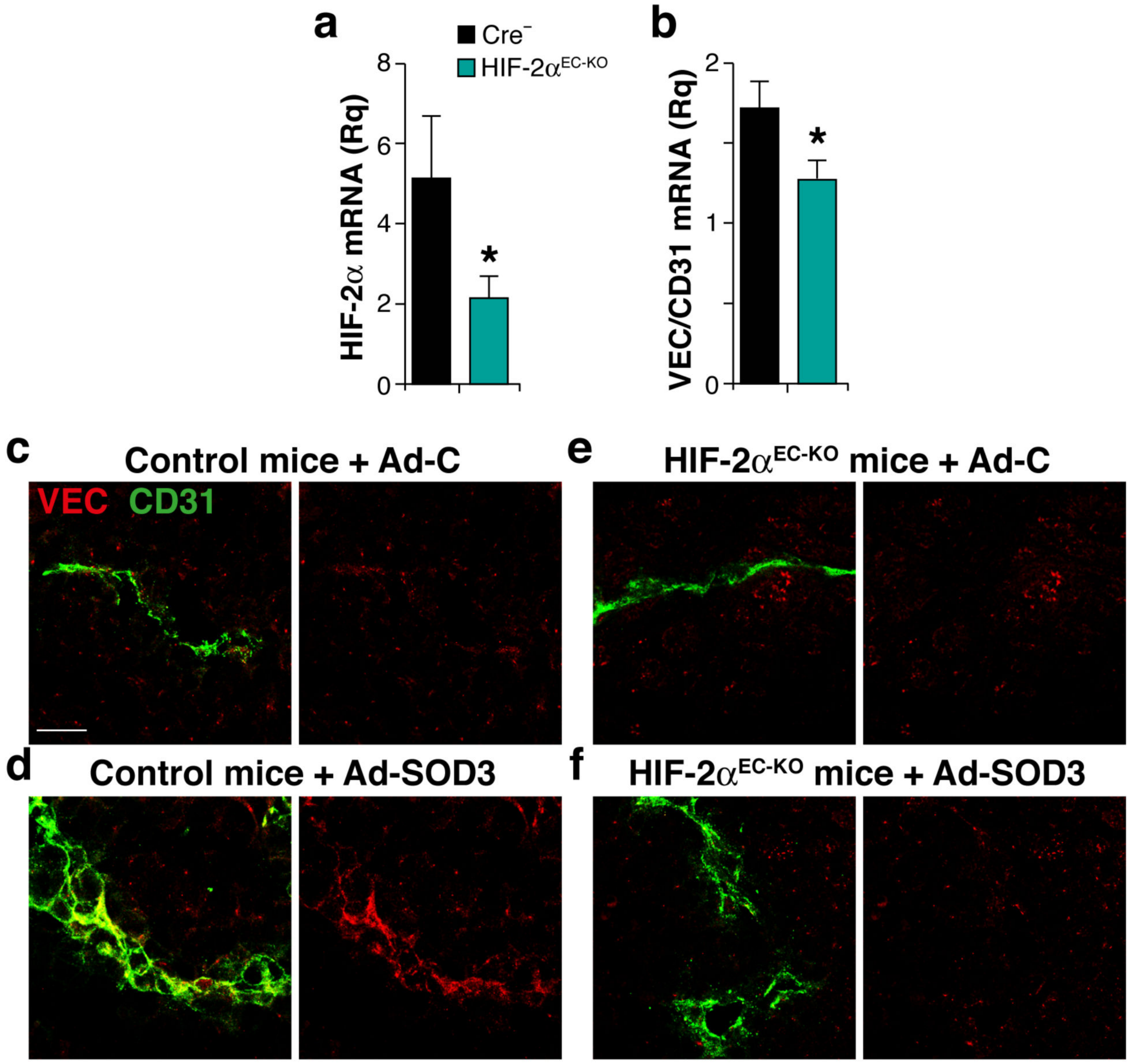

Supplementary Figure 10. HIF-2α mediates SOD3-induced VEC transcription in vivo. (**a, b**) Relative HIF-2α and VEC mRNA levels in EC isolated from tumors grafted in control (Cre-) and HIF-2α$^{EC\text{-}KO}$ mice. Data shown as mean ± SEM of triplicates from 4 mice/genotype. * $p < 0.05$, two-tailed Student's *t*-test. (**c-f**) Immunofluorescence images of VEC (red) and CD31 (green) staining in tumors from Ad-C- and Ad-mSOD3-treated control (Cre-) and HIF-2α$^{EC\text{-}KO}$ mice. Bar, 20 μm.

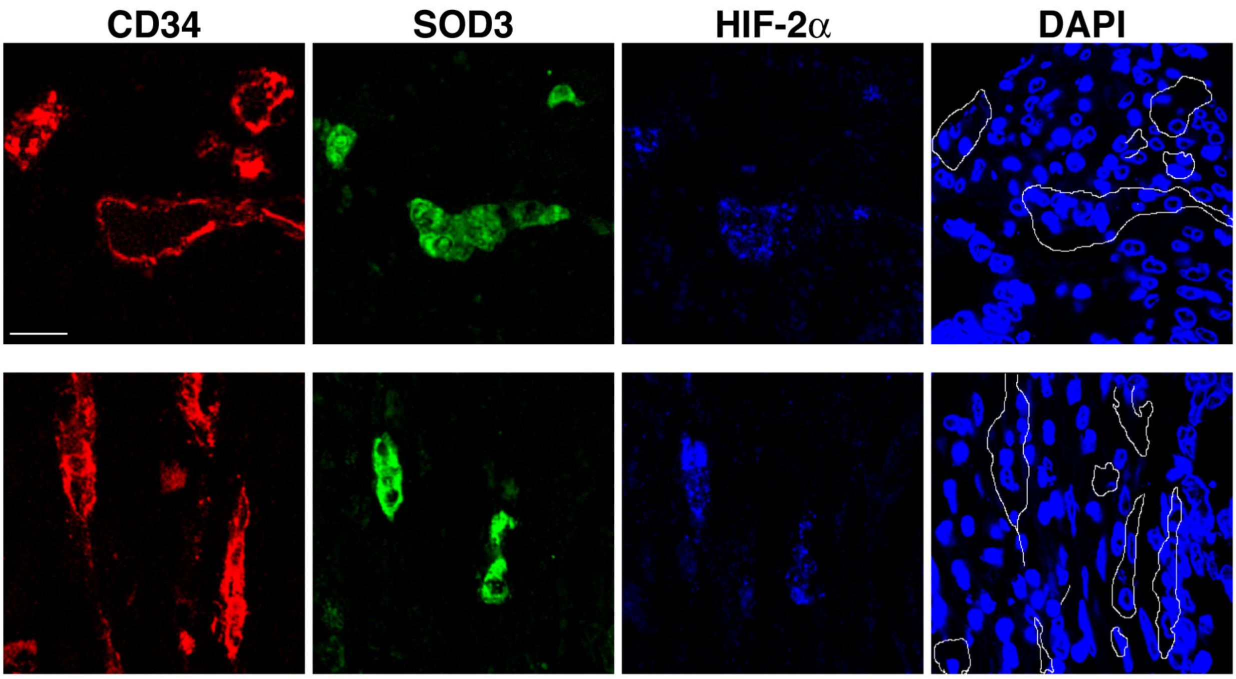

Supplementary Figure 11. Coregulation of SOD3, VEC and HIF-2α in primary CRC tumors. Single-color images and DAPI counterstaining of the micrographs in Fig. 8j and 8k. White lines in the DAPI panel represent the contour of CD34-stained areas. Bar, 20 μm.

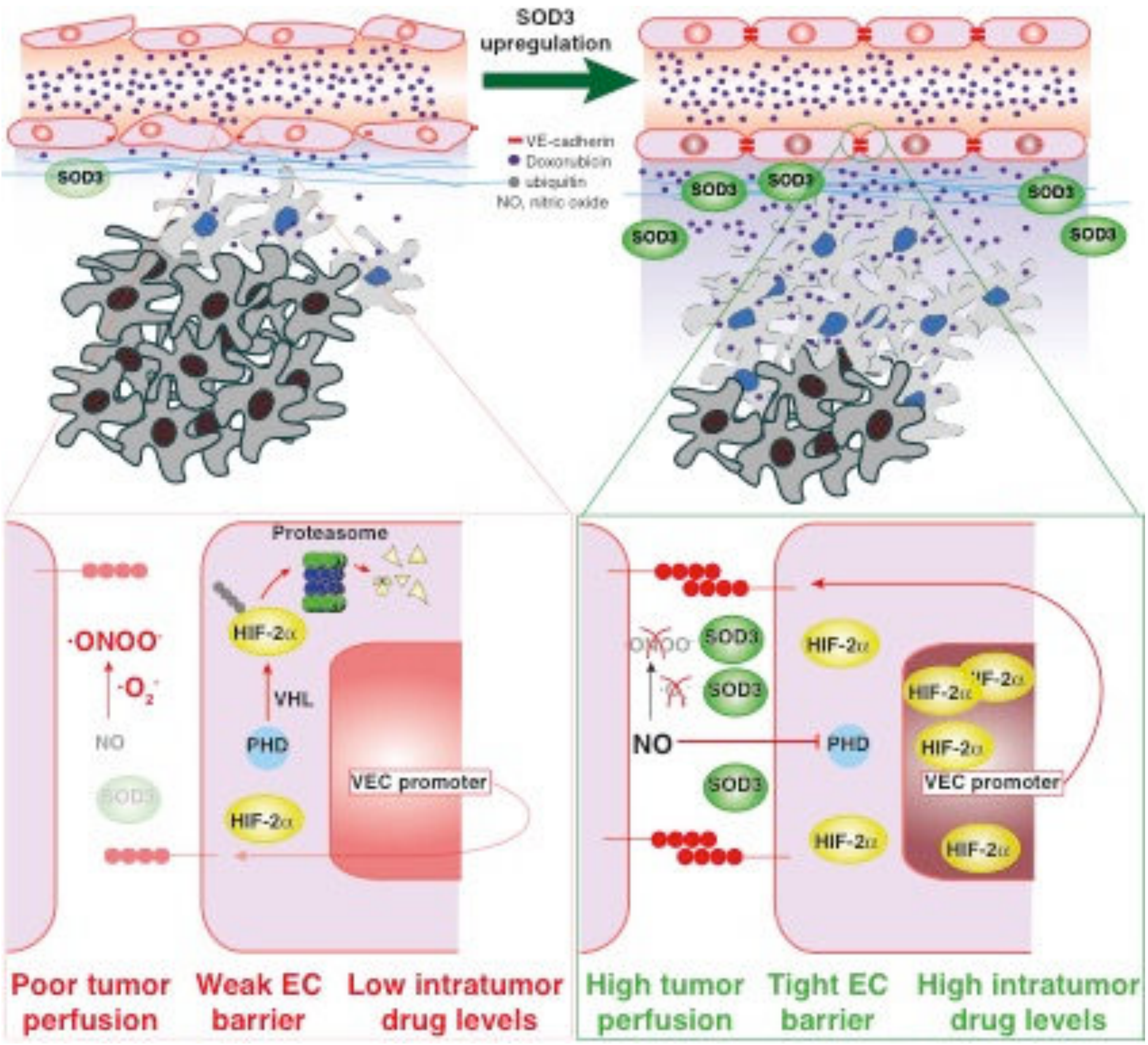

Supplementary Figure 12. Proposed mechanism by which SOD3 increases Doxo delivery into tumors. Growing tumors express low levels of SOD3 (left), which causes high superoxide anion (·O2$^-$) levels and NO oxidation. In these conditions, PHDs hydroxylate HIF-2α, leading to its ubiquitination and degradation. Genetic or pharmacological upregulation of SOD3 near endothelial cells (right) prevents NO oxidation, which enables NO-mediated inhibition of PHD activity. HIF-2α is then stabilized, which enhances its binding to the VEC promoter, increases VEC transcription and strengthens the endothelial barrier function. This normalizes tumor vasculature and promotes the transport of small molecules such as Doxo into the tumor mass, thus improving the tumor response to chemotherapy.

Supplementary Figure 13. Full blots for main figures.

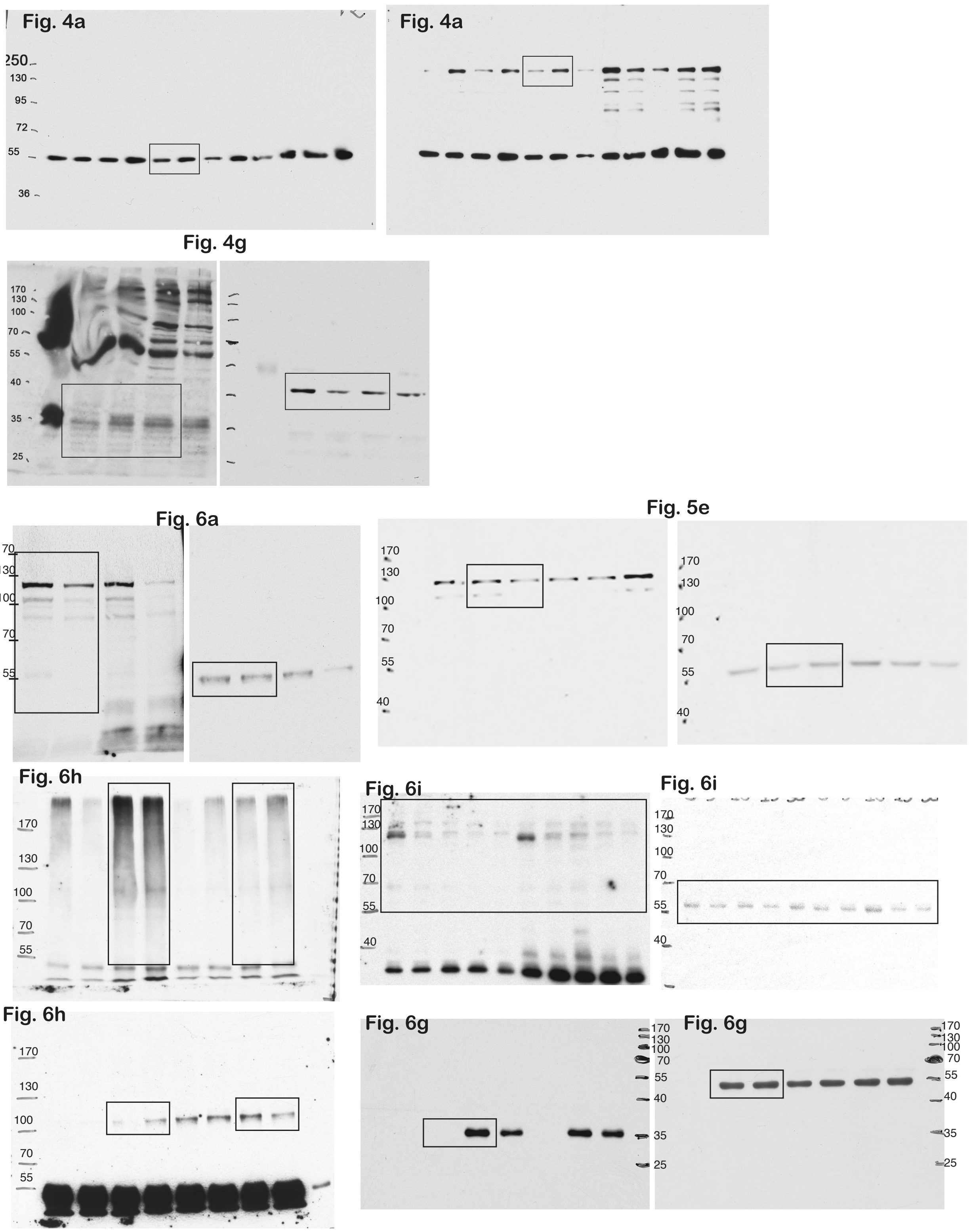

Supplementary Figure 14. Full blots for supplementary figures

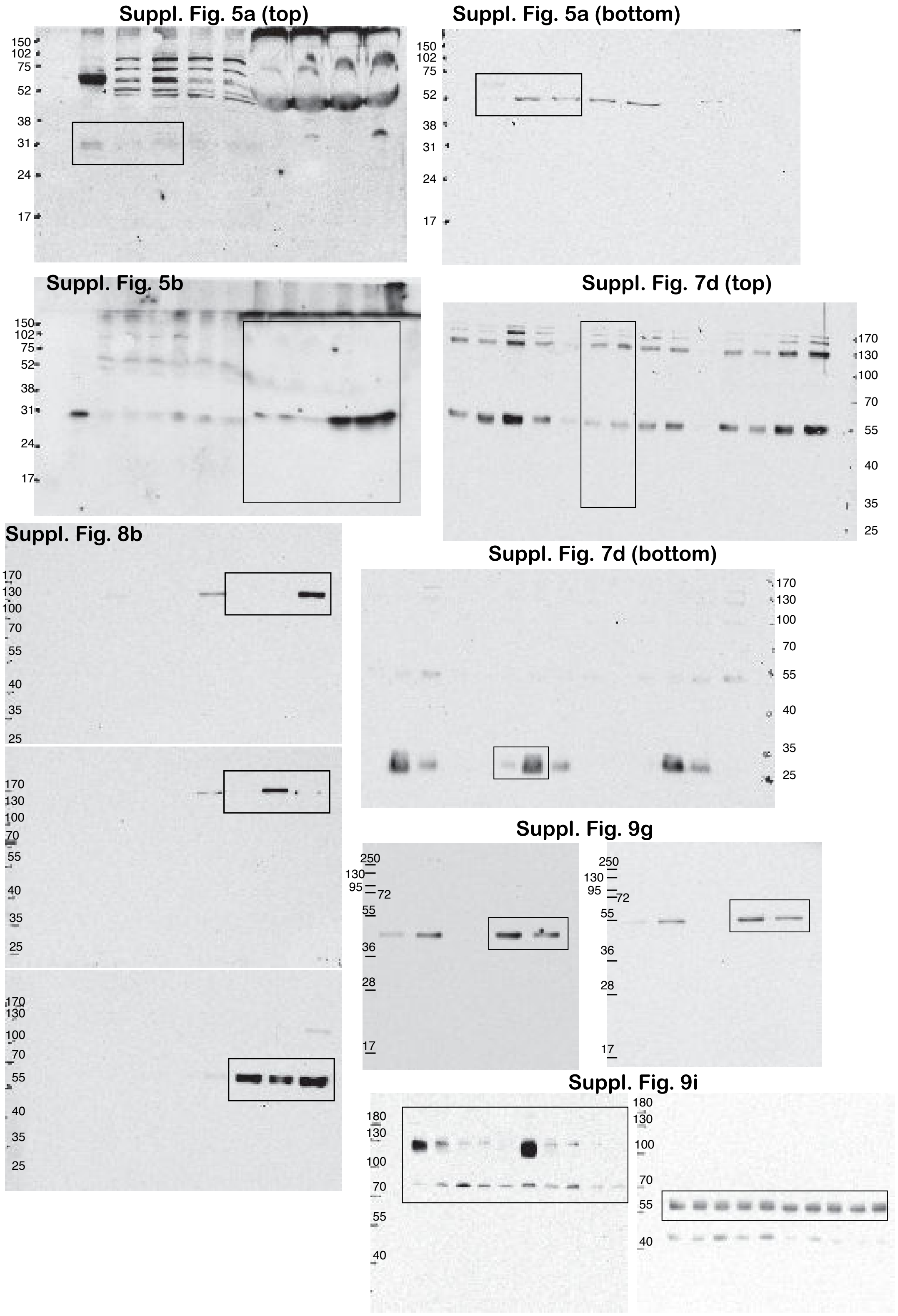

Supplementary Table 1. Primers used for RT-qPCR

Gene	Fw. (5'-3')	Rev (5'-3')
**Mouse SOD3**	GGGGAGGCAACTCAGAGG	CCAACATGGCTGAGGTTCTC
**Human SOD3**	GGTGCAGCTCTCTTTTCAGG	AACACAGTAGCGCCAGCAT
**Mouse VEC**	GTTCAAGTTTGCCCTGAAGAA	GTGATGTTGGCGGTGTTGT
**Human VEC**	GCAGTCCAACGGAACAGAA	CATGAGCCTCTGCATCTTCC
**Mouse HIF-2α**	CTCCAGGAGCTCAAAAGGTG	CAGGTAAGGCTCGAACGATG
**Mouse HIF-1α**	GGGTACAAGAAACCACCCAT	GAGGCTGTGTCGACTGAGAA
**Mouse CD31**	AGGCTTGCATAGAGCTCCAG	TTCTTGGTTTCCAGCTATGG
**Mouse Dll4**	AGGTGCCACTTCGGTTACAC	GGGAGAGCAAATGGCTGATA
**Mouse eNOS**	CCAGTGCCCTGCTTCATC	GCAGGGCAAGTTAGGATCAG
**Mouse Flt1**	GGCCCGGGATATTTATAAGAAC	CCATCCATTTTAGGGGAAGTC
**Mouse ICAM1**	GTGGCGGGAAAGTTCCTG	CGTCTTGCAGGTCATCTTAGGA
**Mouse Notch1**	GGATGCTGACTGCATGGAT	AATCATGAGGGGTGTGAAGC
**Mouse Notch2**	CCATTTCAAGTGTTCGTGTCC	CACATTCATCGATGTTCTCTTC
**Mouse Notch4**	TGCTCAACGGCTTCCAGT	TCGTCCATGTCTTTCTCACATC
**Mouse Robo4**	GACCTATATGTGTATGGCCACC	CTCTAGATGTTCCTTGTGGTCC
**Mouse Tie2**	GATTTTGGATTGTCCCGAGGT CAAG	CACCAATATCTGGGCAAATGATG G
**Mouse VEGF-A**	GCAGCTTGAGTTAAACGAACG	GGTTCCCGAAACCCTGAG
**Mouse VCAM1**	TCTTACCTGTGCGCTGTGAC	ACTGGATCTTCAGGGAATGAGT
**Mouse β-actin**	GGCACCACACCTTCTACAATG	TGGATGGCTACGTACATGGCTG
**Human β-actin**	CCCAGCACAATGAAGATCAA	CGATCCACACGGAGTACTTG
**Mouse GAPDH**	AGGTCGGTGTGAACGGATTTG	TGTAGACCATGTAGTTGAGGTC
**Human RPL10A**	CTTCCCTGCTCACACACAAC	CCAACAGCTACAGCCAGACA
